# Knowledge of chronic kidney disease among undergraduate and postgraduate students in a public university in Klang Valley, Malaysia: A cross sectional study

**DOI:** 10.1371/journal.pone.0274038

**Published:** 2022-09-23

**Authors:** Lean Cheong Loo, Kah Wei Ong, Aida Khalisha Ahmad Nidzwal, Muhammad Helmi Razali, Nizal Ahmad, Azlinah Naim, Faiz Daud, Abdul Halim Abdul Gafor, Norfazilah Ahmad

**Affiliations:** 1 Department of Community Health, Faculty of Medicine, Universiti Kebangsaan Malaysia Medical Centre, Cheras, Kuala Lumpur, Malaysia; 2 Department of Medicine, Faculty of Medicine, Universiti Kebangsaan Malaysia Medical Centre, Cheras, Kuala Lumpur, Malaysia; Faculty of Health Sciences - Universidade da Beira Interior, PORTUGAL

## Abstract

The prevalence of chronic kidney disease (CKD) risk factors such as diabetes mellitus, hypertension, and obesity among the young Malaysians are increasing. Understanding the factors associated with CKD knowledge could assists healthcare providers to design health education programmes. There are scarce local studies on CKD knowledge and its associated factors among university students. This subpopulation comprises of young people with diverse background and characteristics. This study was aimed to assess the CKD knowledge and its associated factors among university students. A cross-sectional study was conducted among Universiti Kebangsaan Malaysia students from July 2020 to August 2020. A convenience sampling method was applied. All students were invited to complete an online survey using Google Forms that were sent to their email. The survey consisted of questions related to their sociodemographic, socioeconomics, university programme enrolled, medical history, lifestyle characteristics and CKD knowledge. The data were analysed using SPSS Statistics 26.0. Multiple logistic regression analysis was performed to identify the final associated factors after controlling for confounders. A total of 3074 students participated and 32.6% of them had below average CKD knowledge. Students of male gender, enrolment in undergraduate programmes and non-health-related faculties/institutes were more likely to have below average CKD knowledge. Students who are Chinese, from high monthly household income family and with family history of CKD were less likely to have below average CKD knowledge. Almost one-third of the students had below average CKD knowledge. The six associated factors with CKD knowledge were non-modifiable. Of the six factors, three were associated with students being more likely to have below average CKD knowledge; another three were associated with students being less likely to have below average CKD knowledge. Future health education programmes to enhance CKD knowledge should be designed focusing on students who are more likely to have below average CKD knowledge.

## Introduction

Chronic kidney disease (CKD) is defined as abnormalities of kidney structure or function, that are present for > 3 months, and present health implications. CKD is classified based on the cause, estimated glomerular filtration rate (eGFR), and albuminuria category [1]. CKD can be categorized into five stages, namely: stage 1 (eGFR >90 ml/min/1.73 m^2^ and persistent albuminuria), stage 2 (eGFR 60–89 ml/min/1.73 m^2^ and persistent albuminuria), stage 3 (eGFR 30–59 ml/min/1.73 m^2^), stage 4 (eGFR 15–29 ml/min/1.73 m^2^), and stage 5 (eGFR <15 ml/min/1.73 m^2^). CKD is an important global health problem as its prevalence and global burden are rising rapidly. Worldwide, approximately 200 million people have CKD [2] and approximately 1.4 million deaths resulted from it in 2019. The global all-age prevalence of CKD has increased by 29.3% since 1990 and resulted in 35.8 million disability-adjusted life-years in 2017 [3]. CKD was also estimated to become the fifth most common cause of years of life lost worldwide by 2040 [4, 5].

In Malaysia, CKD prevalence increased from 9.1% in 2011 [6] to 15.5% in 2017 [7]. Local studies have shown that CKD has detrimental impacts on macroeconomics [8, 9] and health-related quality of life [10, 11]. Previous non-local studies [12, 13] identified associated non-modifiable factors with CKD such as age, gender, education level and personal income. These studies [12–14] also demonstrated the modifiable factors associated with CKD which included alcohol consumption, overweight, obesity, prediabetes, diabetes mellitus and hypertension. Studies conducted in Malaysia also examined the non-modifiable [15], modifiable [7] factors and the interactions between these factors [16] with CKD. One modifiable factor is knowledge, and it is agreed that knowledge is a crucial step towards preventing a disease [17, 18].

A person who is knowledgeable and aware of CKD would tend to adopt a healthy lifestyle, which could lower the risk of CKD [19]. Early-stage CKD is typically asymptomatic, possibly resulting in low awareness among at-risk individuals and in people with CKD. A United States study indicated that only a small proportion of people identified with CKD at a health screening programme, were made aware of their diagnosis [20], while a national survey indicated that only 4% of respondents with CKD were aware of their diagnosis [6].

CKD can affect any age group, including the young. The prevalence of CKD risk factors, especially diabetes mellitus and hypertension among young adults in Malaysia has increased throughout the years [21]. Among the Southeast Asian countries, Malaysia recorded among the highest level of obesity, which is another CKD risk factor [22]. Malaysians have become increasingly obese in recent years [23]. Furthermore, young Malaysians are overweight and obese due to alteration in their eating and activity habits [24].

University students are a segment of the general population wherein the majority are young adults. This subpopulation comprises people with diverse sociodemographic, and socioeconomic characteristics and university programme enrolment. Some university students join health- or non-health related programmes that might or might not provide detail information on CKD. Regardless, university students need to be equipped with such knowledge, to understand the seriousness of CKD and its implications to not only for themselves but also their family members. The adaptation of a healthy lifestyle in early years would likely be maintained throughout a person’s lifetime [25] and would not only benefit the person but also generate a positive social transformation for a better society. Hence, it is extremely crucial for the young population to acquire adequate knowledge on CKD to prevent this disease.

University students in Rwanda, East Africa, showed poor knowledge on CKD risk factors and preventive measures [26]. Furthermore, students studying health-related subjects and who may have more knowledge than the general or overall student population showed an overall lack of knowledge on CKD presentation, progression and complications [27, 28]. In Malaysia, there are scarce local studies investigating knowledge of CKD and its associated factors. Previously, three local studies focused on general [6], hospital based [17] and university students (enrolled in undergraduate and health-related programmes only) populations [17, 28]. These studies only involved descriptive [6] or bivariable analyses [17, 28] without controlling for possible confounders.

More studies should be conducted as Malaysia is a multi-ethnic country with highly diverse sociocultural characteristics. Such studies could aid recognition of the knowledge gap in certain sub-population, who could be disproportionately affected by CKD, based on factors such as age and ethnicity [27]. Therefore, the present study was aimed at measuring the knowledge of CKD and its associated factors among students from both undergraduate and postgraduate programmes across different courses at a public university in Klang Valley, Malaysia.

## Materials and methods

### Study setting and population

A cross-sectional study was conducted among undergraduate and postgraduate students of Universiti Kebangsaan Malaysia (UKM) from July to August 2020 to assess CKD knowledge and its associated factors. UKM is a public university which was established in 1970 and has since become a reputable research public university [29]. The main campus is in Bandar Baru Bangi, which is south of Malaysia’s capital, Kuala Lumpur. UKM also has a health-related campus in Kuala Lumpur that houses the Faculty of Health Sciences, Faculty of Dentistry and Faculty of Pharmacy. UKM also has its own teaching hospital in Cheras, Kuala Lumpur, with an adjoining Faculty of Medicine and the UKM Medical Molecular Biology Institute.

A convenience sampling method was applied in which students were invited to participate in the study via email; their email addresses were provided by the UKM Centre of Academic Management. The current study adhered to the tenets of the Declaration of Helsinki. Study approval was obtained from the UKM Research Ethics Committee (research code: FF-2020-271). The inclusion criteria were Malaysian citizens, aged ≥18 year and full-time students. The exclusion criterion was self-reported history of CKD. The sample size was calculated using PS software [30] at 5% significance level, study power of 80% and with reference to a previous study [19] which assessed the CKD knowledge among primary outpatients measured by the questionnaire used in this study. The authors reported that patients with and without comorbidities, had below average CKD knowledge of 47.9% and 39.2%, respectively. Thus, the present study required a minimum sample size of 1260 with missing value of additional 20%.

### Data collection

The students were invited to participate in July–August 2020. A set of questionnaire prepared in Google Forms was distributed via the students’ email addresses. Informed consent was provided in the first section of the online survey form where they are fully informed as to the intent and purpose of the study. The written informed consent has been waived by the UKM Research Ethics Committee. No minors were involved in this study. Students who agree to have their data used for the study, can click an “I Agree” button and have their data submitted online. Students who do not agree to have their data used in the study, can click an “I Do Not Agree” button and their data is not submitted and collected online. We opted to use this method because the study was conducted when Malaysia was under a Movement Control Order following the coronavirus disease 2019 (COVID-19) pandemic outbreak. Students were prohibited from being on-campus to curb the pandemic, and all teaching and learning sessions were conducted online. The questionnaire consisted of two sections: Section A, which assessed the student’s sociodemographic, socioeconomics, university programme enrolled, medical history and smoking status.

Section B consisted of a validated CKD knowledge questionnaire from a study from Singapore [19]. Approval to use the questionnaire was obtained from the author. The questionnaire items had been developed through focus group discussions with members of the public. The questionnaire had been tested with face validity and checked for content saturation. Furthermore, content validity had been through focus group discussions and reviewed by experts (nephrologists and primary care physicians) [19]. Nevertheless, the author did not discuss the construct validity or reliability analyses. The questionnaire consisted of seven questions for assessing the student’s knowledge on CKD, and involved the following domains: i) anatomy, ii) physiology, iii) aetiology, iv) presentation of early CKD symptoms, v) progression, vi) resources available for CKD patients, and vii) treatment available for end-stage renal failure. Students were required to select the best answer for each of the following questions:

1. Anatomy: How many healthy kidney(s) does a person need to lead a normal life? (correct answer: one)
2. Physiology: What is the function of a kidney in the human body? (correct answer: to filter waste products in the blood)
3. Aetiology: What can cause kidney disease? (correct answer: hypertension, diabetes and inherited conditions)
4. Presentation: What are the symptoms of early kidney disease that might progress to kidney failure? (correct answer: can present without any symptoms/ complaints)
5. Progression: Which of the following statement(s) about kidney disease is incorrect? (correct answer: identified that CKD cannot be cured with medication)
6. Resources available: Where can dialysis treatment be carried out? (correct answer: patient can undergo dialysis either at a dialysis centre or at home)
7. Treatment: What is the best medical treatment for end-stage kidney failure? (correct answer: kidney transplant)

Each correct answer was awarded 1 point; incorrect answers or ‘I don’t know’ were awarded 0 points, yielding a maximum score of 7 points and a minimum score of 0 points.

### Study variables

The study outcome was below average knowledge, which was defined as a score for <4/7 correct answers. Average CKD knowledge was considered a score for ≥4/7 correct answers [19]. The sociodemographic characteristics were age (years) and gender (male and female), ethnicity (Malay, Chinese, Indian, others). The socioeconomic characteristics were working status (working and not working), monthly household income [Bottom 40 (B40) group: ≤Malaysian Ringgit (MYR) 4849; Middle 40 (M40) group: MYR 4850–10,959; Top 20 (T20) group: ≥MYR 10,960] [31], marital status (single, married, separated/divorced/widowed) and presence of children (yes or no).

University programme enrolled comprised programme level (undergraduate or postgraduate), faculty/institute (any of the 15 faculties or 12 institutes and recategorized into health and non-health-related faculties/institutes) and years of study. Medical history included personal medical history (history of diabetes mellitus, hypertension or any other chronic medical illness, or none) and family history of chronic illness (family with diabetes mellitus, hypertension or CKD, or none). Smoking status was assessed by asking ‘Have you ever smoked (e.g., cigarette, vape, cigars, pipes, shisha, etc.)’ and was categorized as yes or no.

### Statistical analysis

The data were analysed using SPSS Statistics 26.0 (IBM Corp., Armonk, NY, USA). Categorical data were described as the frequency (*n*) and percentage (%). Normally distributed data were described using the mean and standard deviation (SD). The crude odds ratio (COR) and its corresponding 95% confidence interval (CI) were determined using simple logistic regression. Adjusted analysis of multiple logistic regression analysis (forward likelihood ratio) was performed to identify the adjusted OR (AOR) of the final factors associated with below average CKD knowledge after controlling for possible confounders. The final model was tested for all possible two-way interactions (multiplicative interaction) between the independent variables, and its fitness was assessed. Statistical significance was set at *p* < 0.05.

## Results

### Students’ characteristics

We sent a total of 27 372 emails to the students. Of the recipients, 3109 students responded and completed the survey, yielding a response rate of 11.4%. A total of 35 students were excluded due to self-reported CKD, resulting in 3704 student responses available for analysis. The mean age was 26.34 (SD 6.77) years. The majority of the students were young adults (age ≤ 39 years, 94.2%). The majority were female (76.4%) and Malay (72.5%). Most of the respondents (70.5%) were not working and 55.2% were in the B40 monthly household income group. A total of 80.6% and 85.3% were single and did not have children, respectively. Almost two-thirds of the students (62.5%) were undergraduates at non-health-related faculties/institutes (69.9%). Most of the students (86.7%) did not have any personal medical history, family members with chronic illnesses (73.8%) and non-smokers (91.5%). Table 1 shows the characteristics of the university students.

**Table 1:** Characteristic of the university students attending a public university in Klang Valley, Malaysia (*n* = 3074).

Factors		*n*	*%*	Mean (SD)
**Sociodemographic**				
Age (years)				26.34 (6.77)
	18–19	13	0.4	
	20–21	664	21.6	
	22–24	1114	36.2	
	25–29	526	17.1	
	30–34	336	10.9	
	≥ 35	421	13.8	
Gender				
	Female	2348	76.4	
	Male	726	23.6	
Ethnicity				
	Malay	2229	72.5	
	Chinese	441	14.3	
	Indian	245	8	
	Others	159	5.2	
**Socioeconomic**		*** ***	*** ***	*** ***
Working status				
	Not working	2166	70.5	
	Working	908	29.5	
Monthly household income				
	B40	1696	55.2	
	M40	1151	37.4	
	T20	227	7.4	
Marital status				
	Single	2479	80.6	
	Married	572	18.6	
	Separated/Divorce/ Widowed	23	0.8	
Presence of children				
	Yes	451	14.7	
	No	2623	85.3	
**University programme enrolled**		*** ***	*** ***	*** ***
Programme level				
	Postgraduate	1152	37.5	
	Undergraduate	1922	62.5	
Faculty/ Institutes				
	Health related	925	30.1	
	Non-health related	2149	69.9	
Years of study				2.47 (1.20)
	1	804	26.2	
	2	895	29	
	3	684	22.3	
	4	518	16.9	
	5	173	5.6	
**Medical history & smoking status**		*** ***	*** ***	*** ***
Personal medical history				
	None	2666	86.7	
	Hypertension	102	3.4	
	Diabetes Mellitus	57	1.9	
	Others	249	8	
Family history of chronic illness				
	None	806	26.2	
	Hypertension	1110	36.1	
	Diabetes Mellitus	913	29.7	
	CKD	245	8	
Smoking status				
	No	2814	91.5	
	Yes	260	8.5	

### Knowledge of a public university students about CKD

**Fig 1:**
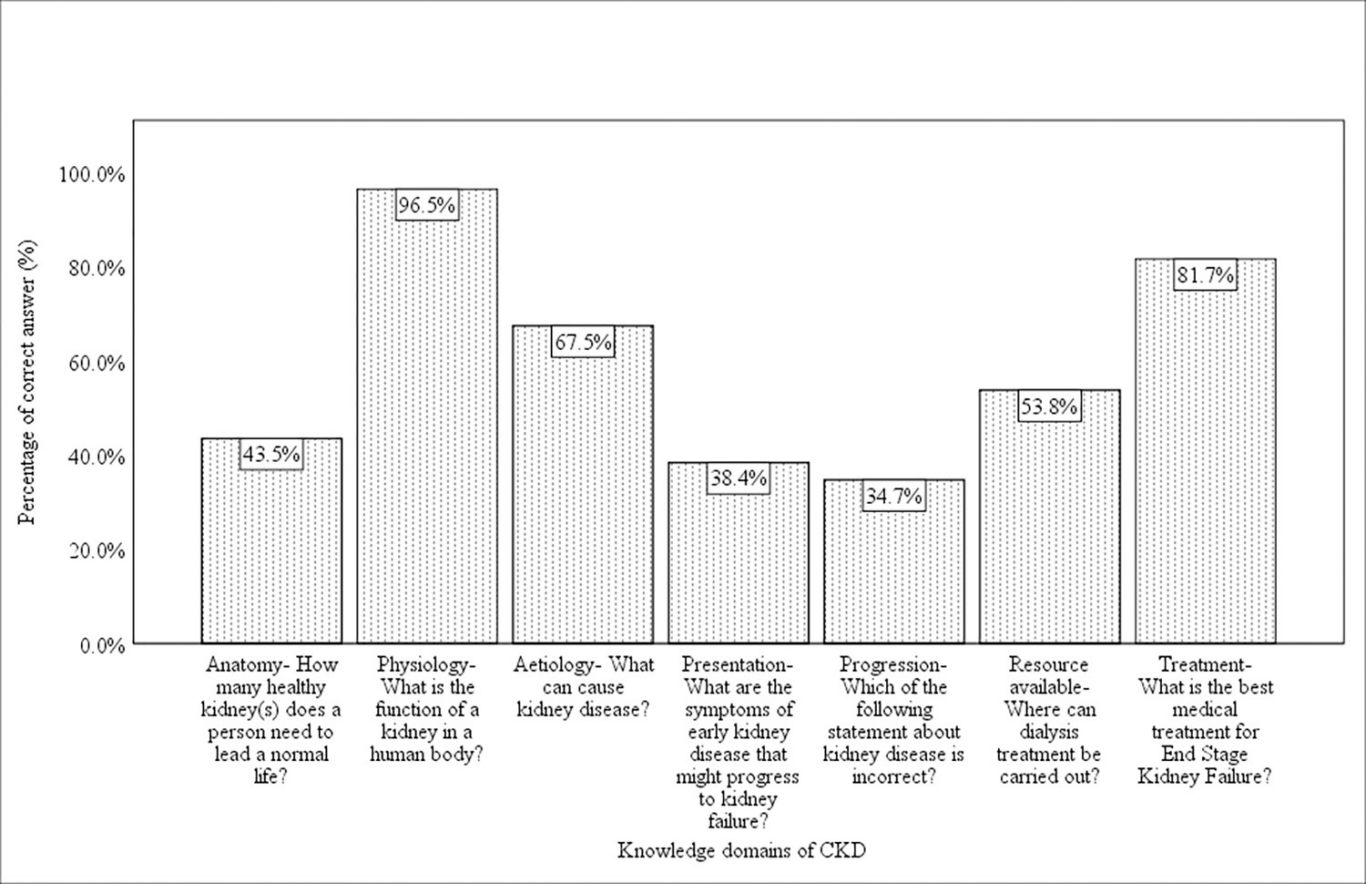
Percentage of correct answers by knowledge domains of CKD.

Out of the 3074 students, 32.6% (95% CI: 31%-34.3%) had below average CKD knowledge. Approximately half of the respondents answered correctly for knowledge on anatomy of the kidney (43.5%). Most of the students answered correctly for knowledge on kidney physiology or function (96.5%) and CKD aetiology (67.5%). However, approximately one-third of the students answered correctly for CKD symptom presentation (38.4%) and knowledge on CKD progression (34.7%). More than half of the students (53.8%) answered correctly for knowledge of resources of dialysis treatment centres. Most of the students correctly answer the best treatment for end-stage renal failure (81.7%). (Fig 1)

### Factors associated with below average knowledge of CKD

Table 2 depicts the simple logistic regression analysis, where nine factors were associated with below average CKD knowledge. Further adjusted analysis (multiple logistic regression) to control for possible confounders showed that only six factors remained significantly associated with below average CKD knowledge (Table 3). Of the six factors, three were associated with students being more likely to have below average CKD knowledge; another three were associated with students being less likely to have below average CKD knowledge. Male students were more likely, while Chinese students were less likely to have below average CKD knowledge. Students from high monthly household income families, were less likely to have below average CKD knowledge. Students enrolled in undergraduate programmes and at non-health-related faculties/institutes were more likely to have below average CKD knowledge. Students with a family history of CKD were less likely to have below average CKD knowledge.

**Table 2:** Preliminary factors associated with CKD knowledge among students of a public university in Klang Valley, Malaysia.

Factors		Below Average CKD Knowledge		Average CKD Knowledge		COR	χ^2^ (df)	*p-*value
		(*n* = 1003)		(*n* = 2071)		(95% CI)		* *
		32.6%		67.4%				* *
Sociodemographic	* *	*n*	*%*	*n*	*%*			
Age (years)	*** ***	-		***-***	*** ***	0.98 (0.97, 0.99)	7.81 (1)	**0.005**
Gender								** **
	Female	735	31.3	1613	68.7	1		
	Male	268	36.9	458	63.1	1.28 (1.08, 1.53)	7.92 (1)	**0.005**
Ethnicity								** **
	Malay	825	37	1404	63	1		** **
	Chinese	53	12	388	88	0.23 (0.17, 0.31)	91.09 (1)	**<0.001**
	Indian	77	31.4	168	68.6	0.78 (0.59, 1.04)	2.96 (1)	0.085
	Others	48	30.2	111	69.8	0.74 (0.52, 1.04)	2.96 (1)	0.085
**Socioeconomic**								
Working status								
	Not working	705	32.5	1461	67.5	1		
	Working	298	32.8	610	67.2	1.01 (0.86, 1.19)	0.02 (1)	0.884
Monthly household income								
	B40	596	35.1	1100	64.9	1		
	M40	358	31.1	793	68.9	0.83 (0.71, 0.98)	5.01 (1)	**0.025**
	T20	49	21.6	178	78.4	0.51 (0.37, 0.71)	16.03 (1)	**<0.001**
Marital status								
	Single	833	33.6	1646	66.4	1		
	Married	164	28.7	408	71.3	0.79 (0.65, 0.97)	5.12 (1)	**0.024**
	Separated/ Divorce/ Widowed	6	26.1	17	73.9	0.70 (0.27, 1.78)	0.57 (1)	0.450
Presence of children								
	Yes	133	29.5	318	70.5	1		
	No	870	33.2	1753	66.8	1.19 (0.95, 1.48)	2.36 (1)	0.124
**University programme enrolled**								
Programme level								
	Postgraduate	354	30.7	798	69.3	1		
	Undergraduate	649	33.8	1273	66.2	1.15 (0.98, 1.34)	3.02 (1)	0.082
Faculty/ Institutes								
	Health related	104	11.2	821	88.8	1		
	Non-health related	899	41.8	1250		5.68 (5.55, 7.08)	236.59 (1)	**<0.001**
Year of study		-		-		0.90 (0.84, 0.96)	11.08 (1)	**0.001**
**Medical history & smoking status**								** **
Personal medical history								** **
	No	872	32.7	1794	67.3	1		** **
	Yes	131	32.1	277	67.9	0.97 (0.78, 1.22)	0.06 (1)	0.810
Family history of chronic illness								
	None	294	36.5	512	63.5	1		
	Hypertension	386	34.8	724	65.2	0.93 (0.77, 1.12)	0.59 (1)	0.442
	Diabetes Mellitus	310	34	603	66	0.90 (0.73, 1.09)	1.20 (1)	0.274
	CKD	13	5.3	232	94.7	0.10 (0.06, 0.17)	62.54 (1)	**<0.001**
Smoking status								
	No	899	31.9	1915	68.1	1		
	Yes	104	40	156	60	1.42 (1.09, 1.84)	6.97 (1)	**0.008**

**Table 3:** Final factors associated with below average CKD knowledge among students of a public university in Klang Valley, Malaysia.

Factors		AOR (95% CI)	χ^2^ stat. (df)	*p-*value
**Sociodemographic**				
Gender				
	Female	1		
	Male	1.26 (1.05, 1.52)	6.00 (1)	**0.014**
Ethnicity				
	Malay	1		
	Chinese	0.29 (0.21, 0.40)	58.63 (1)	**<0.001**
	Indian	0.84 (0.62, 1.13)	1.37 (1)	0.243
	Others	0.84 (0.58, 1.21)	0.88 (1)	0.350
**Socioeconomic**				
Monthly household income				
	B40	1		
	M40	0.85 (0.72, 1.01)	3.36 (1)	0.067
	T20	0.63 (0.44, 0.90)	6.58(1)	**0.010**
**University programme enrolled**				
Programme level				
	Postgraduate	1		
	Undergraduate	1.41 (1.19, 1.65)	15.61 (1)	**<0.001**
Faculty/ Institutes				
	Health related	1		
	Non-health related	4.02 (3.16, 5.13)	126.29 (1)	**<0.001**
**Medical history**				
Family history of chronic illness				
	No	1		
	Hypertension	0.85 (0.70, 1.04)	2.41 (1)	0.121
	Diabetes mellitus	0.83 (0.67, 1.02)	3.21 (1)	0.073
	CKD	0.30 (0.16, 0.55)	14.92 (1)	**<0.001**

^a^Forward Likelihood Ratio multiple logistic regression was applied. No two-way interaction was detected. Multicollinearity was checked (VIF<10). Holsmer-Lemeshow test *p* = 0.335, Nagelkerke R Square 17.9%, classification table (overall correctly classified percentage = 67.6%).

## Discussion

Despite the low response rate in this study, the sample size was relatively larger than that of a previous local study [28] involving university students. Furthermore, this study was conducted in July 2020 to August 2020 when Malaysia was under a Movement Control Order imposed following the COVID-19 pandemic outbreak. Although all teaching and learning sessions were conducted online and students might have checked their email regularly, their top priority at that point of time might not have been to participate in an online survey. The Movement Control Order caused havoc not for only the health system, but also created the largest known disruption of the national education system and affected students individually. University students are known as a susceptible subpopulation that experiences high levels of anxiety, depression and stress [32]. Therefore, existing mental health problem might have been enhanced during the lockdown, which drastically altered and interrupted their educational endeavours [33].

### Knowledge of CKD

CKD is a common and growing public health problem that requires everyone to have adequate knowledge to reduce its development and progression. Our results indicate that almost one-third of UKM students have below average knowledge of CKD. This is lower than that of a local study that showed that 43.5% of respondents had below average knowledge [28]. In that study, Sowtali et al. suggested that this prevalence is quite high, as their study populations were healthcare undergraduates who are supposed to excel in basic human science [28]. For both studies, the same tool and cut off-point were used, and this difference could be due to the present study recruiting both undergraduate and postgraduate students across courses, while the study by Sowtali et al. involved only undergraduate students in health-related courses. Furthermore, despite using the same tool to measure knowledge, Sowtali et al. measured the originally negative phrase question, positively. Another study among students also showed that 44%, 34% and 22% of students had low, moderate and high knowledge levels, respectively, of CKD risk factors as measured by a 21-item questionnaire [26]. The difference with the present study is due to different tools used for measuring CKD knowledge.

Our study reported a high proportion of correct answers for physiology as compared to a Greek study involving primary school pupils [34]. In that study, the pupils had low knowledge levels on kidney function (36.4%) and the difference was largely due to the study populations. The authors [34] emphasized that this could have been due to the exclusion of the urinary system and kidney functions from the school science syllabus. By contrast, Malaysian students learn this topic in the secondary school basic science syllabus. Interestingly the Greek pupils correctly answered whether a person can survive with one kidney (68.5%) as compared to our study population.

A greater proportion of students in our study correctly identified the risk factors (aetiology) and best medical treatment for end-stage kidney failure in our student’s population as compared to the general populations [19, 35]. This could be due to the level of educational attainment where the general population features a range of education levels. The level of educational attainment was significantly associated with CKD knowledge, especially among the general population [36]. Our study demonstrated that the two least correctly answered domains were disease progression (34.7%) and presentation (38.4%). The present study and two other studies [27, 28] used the same tool for measuring CKD knowledge. Sowtali et al. [28] also indicated that approximately one-third of students had correct knowledge on progression (33.3%). Similarly, a United States study reported that the least correctly answered domains were disease presentation (4.1%) and progression (35.5%) [27].

The low knowledge could be due to health education on disease progression commonly being designed for high-risk groups or patients with CKD [37]. It is crucial to design health education materials for not only the general population, but also specific to certain sub-populations such as young students. Knowledge on disease progression is important, as many people with CKD have no symptoms. Thus, some patients are unaware that they have CKD, and most patients with low CKD stage (e.g., Stage 1 and 2) are in the general population [6, 7]. Furthermore, the progression rate from lower to higher CKD stages is difficult to predict and can occur either slowly or abruptly. Notably, the question on disease progression was the only negatively phrased question. The findings must be interpreted with caution and further assessment should be conducted. Colosi [38] stated that the use of negatively worded questions might, introduce new errors due to confusion. He conducted two surveys simultaneously, one with only positively phrased questions and another with both positive and negative wording, and reported significantly different results.

### Factors associated with below average knowledge of CKD

The present study showed that male students were more likely to have below average CKD knowledge. The below average CKD knowledge among male students is possibly due to sex differences in health information-seeking behaviour, which requires further evaluation. A study among college students in China suggested that male students are less likely than female students to seek health information on self-care, disease prevention and self-medication [39]. Female students are also more likely to utilize the Internet for health information, consult a health or medical professional and verify the health information they find with a health or medical professional [40]. However, this finding is not in agreement with another local study [28]. This incongruent finding could be due to the different measurement scales used for examining the association despite the same tool being used for measuring CKD knowledge, the different student types and the relatively very small sample size of the latter study.

Here, we found that Chinese students and students from high monthly household income families were less likely to have below average CKD knowledge. The association of ethnicity with CKD knowledge is inconsistent with that from a local study [17] and from Singapore [19], despite the same ethnicity categories and tool being used for measuring CKD knowledge. This difference could be due to the different study populations and scale for measuring CKD knowledge. The association between ethnicity and knowledge warrants more investigation, as certain ethnicity is particularly affected by the disease and experience more rapid progression [27]. A lack of knowledge of CKD risk factors and progression may contribute to this disparity.

There is limited information on the association of household family income and CKD knowledge among students for further comparison. However, a review has suggested that a higher household income enables parents to acquire better goods such as healthy diet, books and learning materials for their children to thrive better, including cognitive development and health [41]. Moreover, studies [17, 19] that measured CKD knowledge using the same tool and patients attending primary health outpatient settings showed that the high-income group had higher CKD knowledge scores.

The present study indicates that undergraduate students and students from non-health-related faculties/institutes were more likely to have below average CKD knowledge compared to postgraduate students and those at health-related faculties/institutes. A descriptive study of university students in the United States showed that CKD knowledge was low in this population, particularly among the younger students [27]. We postulated that the below average CKD knowledge among the undergraduate students in the present study is due to low health literacy. Undergraduate students have lower highest educational attainment for university enrolment compared to postgraduate students. The level of educational attainment can influence the health literacy of these students, as seen in other study populations [42, 43].

Apart from the level of educational attainment, the students’ health literacy might have been influenced by their younger age. A Malaysian Health Literacy Survey showed that the Malaysian aged 18–24 years had a higher proportion of limited health literacy compared to Malaysian aged 25–39 years [44]. A Germany study also indicated that respondents in the youngest age group (15–29 years) had lower health literacy scores compared to young adult respondents (30–45 years) [45]. This variation could have been due to the different roles and personal and social obligations of the different age groups. However, these previous studies were conducted among the general populations and these postulations need further exploration as we did not detect any possible interaction of these factors with CKD knowledge.

There is a scarcity of information measuring the association between faculty/institute type with CKD knowledge. Most recent studies have involved students either in health-related [28, 46] or non-health-related [26] faculties/institutes. It is expected that students from non-health-related faculties/institutes were more likely to have below average CKD knowledge, compared to their counterparts, as learning disease pathophysiology is not part of their curriculum. Students from health-related faculties/institutes are future health caregivers and having good CKD knowledge is crucial for managing this complicated health issue. However, it is important to promote and advance CKD knowledge to all students for better disease prevention. We found that students with a family history of CKD was less likely to have below average CKD knowledge. A review paper suggested that having family members, friends, or acquaintances with CKD maybe associated with CKD awareness but has not been fully assessed [47]. While a study from Australia indicated that the study subjects with family history of kidney failure was independently associated with higher knowledge scores [48]. These findings should be interpreted with caution because due to the different tools and scales used for measuring the CKD knowledge or awareness of the study population. Nevertheless, this finding was expected, as knowing a person with kidney disease would indirectly raise one’s awareness of the disease.

The strength of our study included the participation of Malaysian students of varying ethnicities and backgrounds. To our knowledge, this is among the first local studies on CKD knowledge and its associated factors that include both undergraduate and postgraduate students across all courses and programmes. The findings presented were also adjusted for confounders. Furthermore, our findings can be used for formulating future studies. Our study has several limitations. This was a single-centre, cross-sectional study that used non-probability sampling. Causal inferences may not be drawn, and the findings cannot be inferred to the whole student population. Selection bias may be present, where the participating students could be more health conscious and more inclined to answer the questions. There is the possibility of information bias, as the students could have sought the correct answers prior to answering the survey, resulting in overestimation of CKD knowledge in this study.

Despite the involvement of content experts (nephrologists and primary care physicians) in the questionnaire development, the authors suggested that further studies should be performed with a more refined questionnaire [19]. We postulated that this suggestion was based on the fact that, the first two of the seven questions addressed general aspects, namely basic anatomy and normal physiology of the kidney to measure CKD knowledge. These aspects were included as understanding or having knowledge of basic anatomy and function of the kidney enabled the individual to remain updated on possible kidney disease development and complications. This is in line with the definition of CKD (kidney structure or function abnormalities) [1].

Furthermore, the questionnaire validation was not clearly explained. A questionnaire is one of the most common techniques used to measure the knowledge level in a community [49]. A valid and reliable questionnaire must have simple and precise wording, reflect the underlying theory or concept to be measured and able to assess change [50]. Thus, a questionnaire should undergo a development and validation to ensure the reliability of the research findings [49, 50]. Nevertheless, we used this questionnaire after successfully obtaining permission from the author and the questionnaire was developed and used in a community in Singapore. Malaysia and Singapore are ethnically diverse countries, with shared history, cultural roots, and other demographic profiles.

Factors that we hypothesized earlier that would influence CKD knowledge, such as health information-seeking behaviour and health literacy, should be examined further. More studies are needed to explore the types of knowledge (system health knowledge, action-related health knowledge and effectiveness health knowledge), which influence motivational factors (perceived health threat, attitude towards health action, attitude towards health outcome and subjective norms that lead to decisions [51].

Stratified analysis based on programme level and of faculty/institute type should also be investigated as the students have different roles and obligations that might influence their knowledge level. Our study could provide a foundation for intervention strategies for improving the CKD knowledge of this young population. Although CKD prevalence in childhood is uncommon and children were not our focus study population, we hope that our findings highlight the need to design a school education programme on kidney function and the CKD risk factors as early as the primary school level. Such a programme may ensure a healthy and health-literate population starting from a young age [34]. In addition to university students, all young populations should be provided with CKD knowledge. The young population is at a transitional stage during which they develop either healthy or unhealthy habits that will influence their wellbeing throughout adulthood [52]. Based on our findings, specifically empowering youths with knowledge on the symptoms of CKD development and progression should be emphasised. This is a challenge as symptoms do not usually develop in the early stages of the CKD and its progression [1].

## Conclusion

Almost one-third of the students in this study had below average CKD knowledge. The associated factors were non-modifiable and included male gender, Chinese ethnicity, high monthly household income family, undergraduate students, enrolment in non-health-related faculties, and family history of CKD. Future health education programmes or training to enhance CKD knowledge should be designed focusing on students who are likely to have below average CKD knowledge.
